# Supplementary material for: Zoledronate Causes a Systemic Shift of Macrophage Polarization towards M1 In Vivo
Source: Int J Mol Sci. 2021 Jan 28;22(3):1323. doi: 10.3390/ijms22031323 (PMC7865688; doi:10.3390/ijms22031323)
Supplement: Supplementary file 1 [file ijms-22-01323-s001.pdf]

Supplementary tables

Suppl. table S1 CD68, iNOS und CD163 expressions (pos. cells/all cells in %) and iNOS,CD163 and CD68 ratios ((pos. cells/all cells in %)/(pos. cells/all cells in %)) in spleen white pulp specimen of wistar rats depending on BP application and surgical trauma. The values represent the median. The standard deviation (SD) and the p-value (Mann-Whitney-U-Test). Significant p-values are indicated in bold letters.

Marker	n	CD68		iNOS		CD163	
		Median	SD	Median	SD	Median	SD
**BP-application** - all cases							
no	48	1.84	0.38	7.36	4.45	0.18	0.11
yes	44	1.68	0.36	7.99	2.89	0.11	0.07
p-value		0.129		0.167		**<0.001**	
**BP-application** - only no surgical trauma cases							
no	26	1.80	0.35	8.38	3.38	0.14	0.09
yes	25	1.66	0.35	7.71	3.07	0.09	0.05
p-value		0.418		0.665		**0.002**	
**BP-application** - only surgical trauma cases							
no	22	1.88	0.40	5.91	5.47	0.23	0.12
yes	19	1.72	0.37	10.26	2.56	0.12	0.09
p-value		0.25		0.284		**0.004**	
**surgical trauma** - all cases							
no	51	1.73	0.35	7.71	3.21	0.12	0.08
yes	41	1.83	0.39	8.36	4.33	0.18	0.11
p-value		0.364		0.256		**<0.001**	
**surgical trauma** - only no BP application cases							
no	26	1.80	0.35	8.38	3.38	0.14	0.09
yes	22	1.88	0.40	5.91	5.47	0.23	0.12
p-value		0.408		0.591		**0.003**	
**surgical trauma** - only BP application cases							
no	25	1.66	0.35	7.71	3.07	0.09	0.05
yes	19	1.72	0.37	10.26	2.56	0.12	0.09
p-value		0.767		0.121		**0.036**	

Marker	n	Ratio iNOS/CD68		Ratio CD163/CD68		Ratio iNOS/CD163	
		Median	SD	Median	SD	Median	SD
**BP-application** - all cases							
no	48	4.62	2.49	0.10	0.06	52.40	52.63
yes	44	5.35	1.65	0.06	0.05	82.58	116.42
p-value		0.067		**<0.001**		**0.001**	
**BP-application** - only no surgical trauma cases							
no	26	4.91	1.89	0.08	0.04	56.42	56.35
yes	25	4.53	1.91	0.05	0.03	76.15	137.92
p-value		0.44		**0.002**		**0.011**	
**BP-application** - only surgical trauma cases							
no	22	4.20	3.07	0.12	0.08	24.84	47.76
yes	19	5.50	1.21	0.07	0.07	90.76	77.40
p-value		0.136		**0.039**		**0.01**	
**surgical trauma** - all cases							
no	51	4.77	1.90	0.07	0.04	63.53	109.82
yes	41	5.29	2.39	0.11	0.08	60.69	66.72
p-value		0.344		**0.003**		0.163	
**surgical trauma** - only no BP application cases							
no	26	4.91	1.89	0.08	0.04	56.42	56.35
yes	22	4.20	3.07	0.12	0.08	24.84	47.76
p-value		0.983		**0.014**		0.247	
**surgical trauma** - only BP application cases							
no	25	4.53	1.91	0.05	0.03	76.15	137.92
yes	19	5.50	1.21	0.07	0.07	90.76	77.40

p-value	0.132	0.11	0.292

Suppl. table S2 CD68, iNOS und CD163 expressions (pos. cells/all cells in %) and iNOS. CD163 and CD68 ratios ((pos. cells/all cells in %)/(pos. cells/all cells in %)) in skin epithelium specimen of wistar rats depending on BP-application depending on BP-application and surgical trauma. The values represent the median. the standard deviation (SD) and the p-value (Mann-Whitney-U-Test) . Significant p-values are indicated in bold letters.

Marker	n	CD68		iNOS		CD163	
		Median	SD	Median	SD	Median	SD
**BP-application** - all cases							
no	43	0.03	0.08	0.00	0.09	0.05	0.11
yes	33	0.11	0.12	0.00	0.03	0.13	0.12
p-value		**0.001**		0.168		**<0.001**	
**BP-application** - only no surgical trauma cases							
no	21	0.04	0.07	0.00	0.11	0.06	0.08
yes	23	0.12	0.08	0.00	0.03	0.12	0.10
p-value		**0.015**		0.081		**0.017**	
**BP-application** - only surgical trauma cases							
no	22	0.00	0.09	0.00	0.06	0.04	0.14
yes	10	0.09	0.17	0.00	0.03	0.21	0.15
p-value		**0.035**		0.857		**0.006**	
**surgical trauma** - all cases							
no	44	0.08	0.08	0.00	0.08	0.09	0.09
yes	32	0.06	0.13	0.00	0.05	0.09	0.16
p-value		0.493		0.263		0.966	
**surgical trauma** - only no BP application cases							
no	21	0.04	0.07	0.00	0.11	0.06	0.08
yes	22	0.00	0.09	0.00	0.06	0.04	0.14
p-value		0.617		0.132		0.451	
**surgical trauma** - only BP application cases							
no	23	0.12	0.08	0.00	0.03	0.12	0.10
yes	10	0.09	0.17	0.00	0.03	0.21	0.15
p-value		0.893		0.893		**0.031**	

Marker	n	Ratio iNOS/CD68		Ratio CD163/CD68		Ratio iNOS/CD163	
		Median	SD	Median	SD	Median	SD
**BP-application** - all cases							
no	43	0.00	0.32	0.56	1.38	0.00	0.39
yes	33	0.00	0.43	1.10	2.14	0.00	0.34
p-value		0.588		**0.029**		0.18	
**BP-application** - only no surgical trauma cases							
no	21	0.00	0.39	0.59	1.66	0.00	0.29
yes	23	0.00	0.45	1.09	1.40	0.00	0.41
p-value		0.397		0.483		0.276	
**BP-application** - only surgical trauma cases							
no	22	0.00	0.00	0.34	0.64	0.00	0.51
yes	10	0.00	0.41	1.11	3.10	0.00	0.08
p-value		0.73		**0.019**		0.845	
**surgical trauma** - all cases							
no	44	0.00	0.42	1.01	1.49	0.00	0.35
yes	32	0.00	0.29	0.90	2.42	0.00	0.40
p-value		0.119		0.844		0.325	
**surgical trauma** - only no BP application cases							
no	21	0.00	0.39	0.59	1.66	0.00	0.29
yes	22	0.00	0.00	0.34	0.64	0.00	0.51
p-value		0.159		0.336		0.398	
**surgical trauma** - only BP application cases							
no	23	0.00	0.45	1.09	1.40	0.00	0.41
yes	10	0.00	0.41	1.11	3.10	0.00	0.08
p-value		0.885		0.332		0.871	
